# The impact of gender-blindness on social-ecological resilience: The case of a communal pasture in the highlands of Ethiopia

**DOI:** 10.1007/s13280-016-0846-x

**Published:** 2016-11-22

**Authors:** Lemlem Aregu, Ika Darnhofer, Azage Tegegne, Dirk Hoekstra, Maria Wurzinger

**Affiliations:** 1 WorldFish Yangon, West Gyogone, Bayint Naung Road, Insein Township, Yangon, Myanmar; 2 University of Natural Resources and Life Sciences Vienna, Feistmantelstrasse 4, 1180 Vienna, Austria; 3 ILRI, P.O. Box 5689, Addis Ababa, Ethiopia; 4 University of Natural Resources and Life Sciences Vienna, Gregor-Mendel-Strasse 33, 1180 Vienna, Austria

**Keywords:** Knowledge diversity, Livestock management, Natural resource management, Social justice, Social structure

## Abstract

We studied how the failure to take into account gendered roles in the management of a communal pasture can affect the resilience of this social-ecological system. Data were collected using qualitative methods, including focus group discussions, in-depth interviews, and participant observations from one community in the highlands of Ethiopia. The results show that women are excluded from the informal institution that defines the access and use rules which guide the management of the communal pasture. Consequently, women’s knowledge, preferences, and needs are not taken into account. This negatively affects the resilience of the communal pasture in two ways. Firstly, the exclusion of women’s knowledge leads to future adaptation options being overlooked. Secondly, as a result of the failure to address women’s needs, they start to question the legitimacy of the informal institution. The case study thus shows how excluding women, i.e., side-lining their knowledge and needs, weakens social learning and the adaptiveness of the management rules. Being blind to gender-related issues may thus undermine the resilience of a social-ecological system.

## Introduction

As humans shape the natural environment (O’Brien et al. 2009; Warner 2010), social and ecological systems should not be considered in isolation from one another. The concept of social-ecological resilience provides a framework to understand this dynamic and complex interaction between a community and its natural environment. Resilience is defined as the capacity of a system to absorb and adapt to disturbances through a process of reorganization, so as to essentially retain the same function and identity (Holling 1973; Chapin 2009; Folke et al. 2010). While acknowledging that social-ecological resilience is dependent on both ecological and social dynamics, the emphasis in much of the literature is on understanding ecological dynamics and how these are influenced by human activities (Folke et al. 2004). While some scholars have studied the influence of social structure on social-ecological resilience—e.g., through social network analysis (Crona and Hubacek 2010)—only limited attention has been paid to the influence of the social structure of user groups (Meyer and Jepperson 2000). Consequently, how differences between users, e.g., in terms of gender, age, wealth, or ethnicity affect their ability to influence how natural resources are used, is rarely taken into account (Scoones and Cousins 1989; Leach et al. 1999).

Especially in communities whose livelihoods directly depend on natural resources, the social and ecological sub-systems are highly interdependent (Folke et al. 2010). Indeed, social dynamics influence how natural resources are managed, and the work done in feminist political ecology has highlighted how gender relations influence men’s and women’s access to and control over natural resources (Rocheleau and Edmunds 1997; Buechler 2015). Indeed, socially defined gender roles shape and differentiate men’s and women’s tasks, responsibilities, and resources (O’Shaughnessy and Krogman 2011). Feminist political ecologists have also shown how gender relations can shape environmental change and influence ecological dynamics (Agarwal 1997a; Nightingale 2006).

This paper integrates resilience analysis and gender analysis, to increase our understanding of processes that may undermine the ability of a social-ecological system to cope with, to adapt to, and to shape change. Indeed, the resilience concepts point toward the need to cope with both predictable and unexpected change (Berkes and Folke 2002; Folke et al. 2002; Holling 2004; Chapin et al. 2009; Kofinas 2009). In analyzing resilience, it is thus important to understand the mechanisms that may impair change, e.g., by impeding social learning. In this study, social learning is understood as a longitudinal process, which frames the understanding of interrelationships between ecological variables and management practices (Pahl-Wostl et al. 2008). These management practices are understood as being dependent on negotiations between social actors and thus change over time as a response to both ecological and social dynamics. Gender analysis allows to explore the effects of socially defined gender roles on the way in which natural resources are used and managed, and thus the impact of these socially defined roles on the resilience of the social-ecological system. We consider gendered relations as neither deterministic nor static. Rather, they are socially constructed and thus vary across cultures, wealth groups, ethnicity, and even families (Agarwal 1997b). These relations are continuously contested and (re)defined, not least to address changes in the broader context (Agarwal 1997b; Nightingale 2006).

We use a case study in the Ethiopian highlands to illustrate how a lack of attention to gender-based social dynamics can undermine the sustainable management of natural resources. The main objective is to contribute to our understanding of how gender relations drive social dynamics and how these dynamics can influence the choices in the management of a communal pasture.

## Materials and methods

### Site selection and description of the study area

**Fig. 1:**
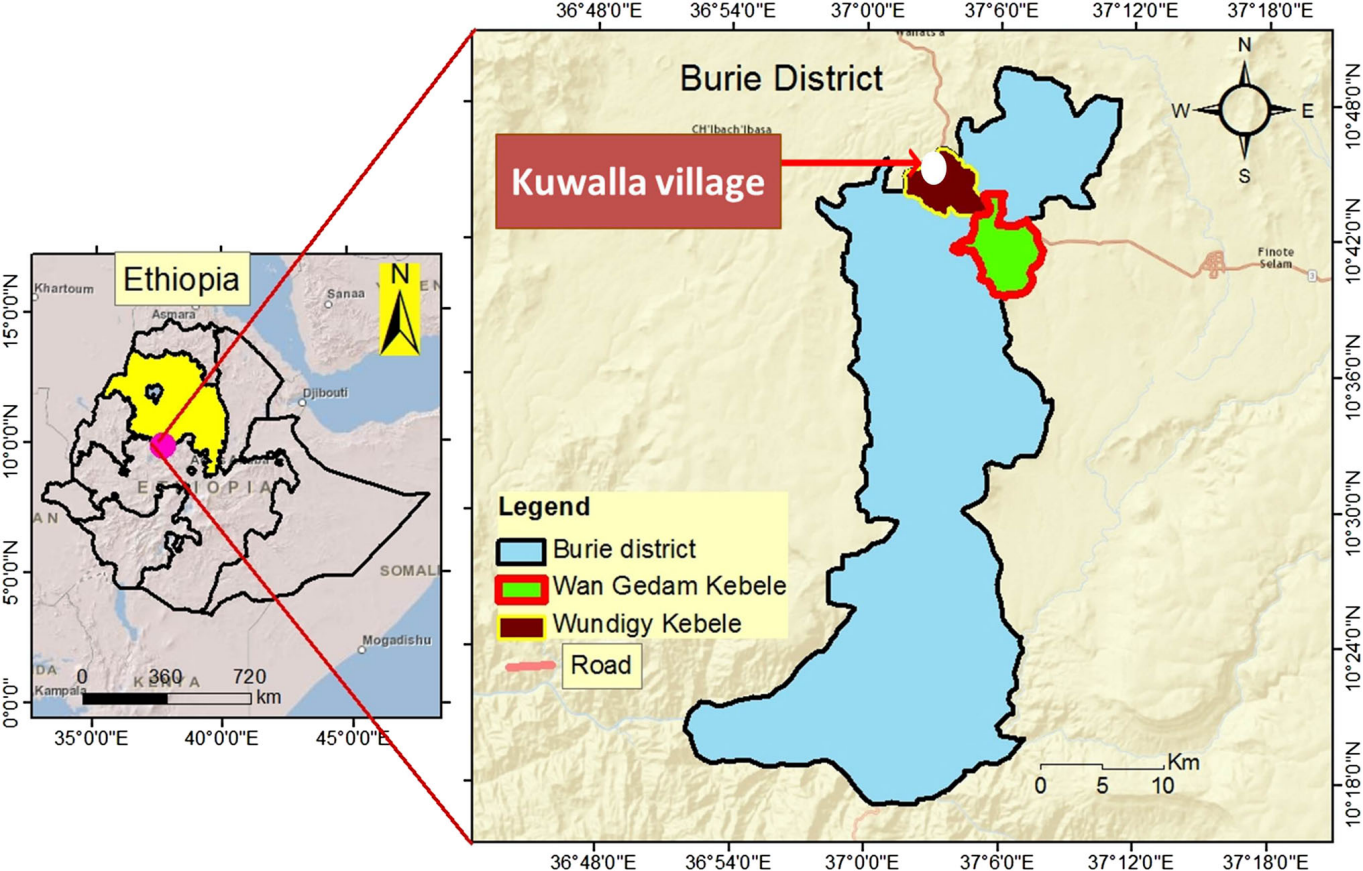
Map of the study site: Physical map of Ethiopia with nine regional states, the Amhara Regional State is highlighted. The enlarged map shows the location of Kuwalla village, in Wundgi *kebele* (the smallest formal local administrative unit), located in Burie district

The data were collected from Kuwalla village, located at an altitude of 2300 m above sea level in the Amhara region (Fig. 1). This village was selected based on a four-step process. It took into account ecological and social indicators of good communal pasture management, which were assessed by a range of officials and experts. Firstly, three officials were asked to suggest potential *kebeles* 1 from Burie District which have a controlled grazing system managed by an informal institution. Secondly, eleven experts from the District Office of Agriculture assessed and rated the 12 potential *kebeles* according to a given set of criteria. These included ecological criteria such as the extent of soil erosion, vegetation cover, and diversity of the species in the pasture, as well as socio-economic criteria such as the number of households and livestock depending on the communal pasture, the heterogeneity of the users, the existence of informal institutions governing the management of the communal pasture, and the number of villages with a controlled grazing system. Thirdly, based on the average rating provided by the experts, the top five *kebeles* were identified and visited for final screening. During the visit, the bio-physical status and the socio-economic importance of the communal pasture were assessed with the assistance of a community representative and development agents. Based on the assessment and after receiving permission by the chairman of the *kebele* to conduct the study, Wundgi *kebele* was selected. Fourthly, out of the 11 villages in Wundgi *kebele* that use a controlled grazing system, Kuwalla village was selected as it had the longest history of managing the communal pasture through controlled grazing system.

**Fig. 2:**
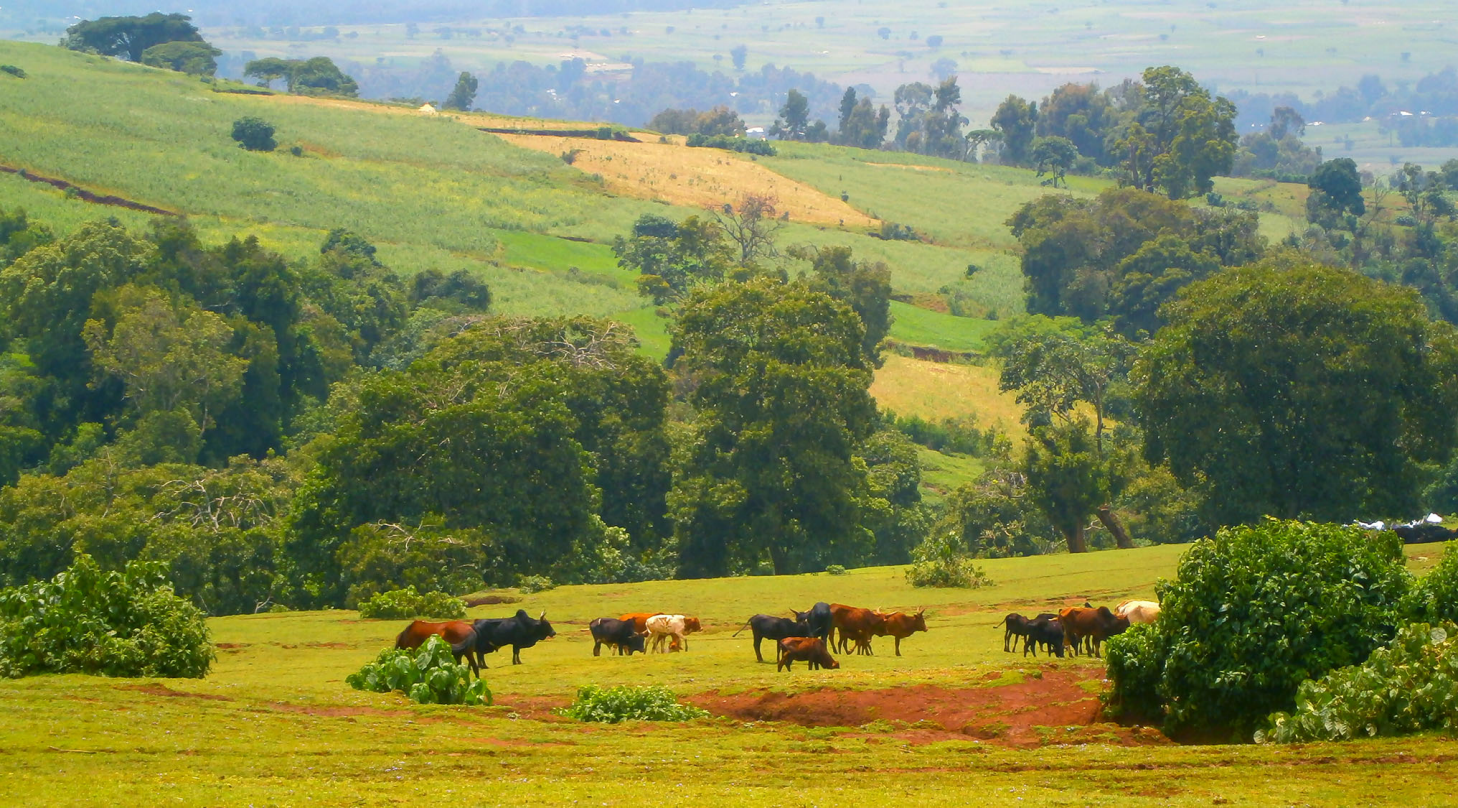
Typical landscape in Burie district during the rainy season. Cattle is grazing on a pasture and the risk of soil erosion due to overgrazing can be seen in the areas with bare soil. Land use is dominated by subsistence farming. Due to population growth, there is an increasing pressure to convert pastures into crop land, which increases the pressure on the remaining pastures. Indeed, in this mixed-crop livestock system, cattle plays an important role as oxen are needed to plough the fields

Kuwalla is characterized by a subsistence mixed farming system, which integrates rain-fed crop cultivation and traditional animal husbandry (Fig. 2). Mixed farming is typical in the Ethiopian highlands and cattle (*Bos indicus*) play an important role as oxen are used to plough fields, while cows produce milk for household consumption, and milk products contribute to income generation. Farmers predominantly produce maize (*Zea mays*), millet (*Eleusine coracana*), *tef* (*Eragrostis abyssinica*), wheat (*Triticum aestivum*), barley (*Hordeum vulgare*), faba bean (*Vicia faba*), field pea (*Pisum sativum*), potato (*Solanum tuberosum*), onion (*Allium cepa*), garlic (*Allium sativum*), cabbage (*Brassica oleracea*), and pepper (*Capsicum* spp.). The crop residues, despite their low nutrient content, make up 50 % of feed for the farm animals. Communal pasture plays a key role as a source of nutritious feed for oxen and cows and contributes 31 % of the total livestock feed. Grass from farm boundaries contributes 11 % and the free grazing area 8 % of the total animal feed.

In the Ethiopian highlands, human population growth leads to a pressure to convert communally managed pastures into individually managed cropland for young farmers. The reduction in available pasture land (Pender and Ehui 2006) and the increase in the number of livestock frequently result in overgrazing, which leads to severe erosion of sloped land without grass cover (Tilahun and Schmidt 2013). Hence maintaining well-managed pastures is crucial. While most communal pastures in the highlands of Ethiopia are accessed freely throughout the year, Kuwalla uses a controlled grazing system. A historical analysis of the Kuwalla communal pasture over the last 40-years revealed that the community implemented a modified version of their traditionally controlled rotational grazing system in response to the negative impact of the open-access system they had between 1975 and 1990 (see Aregu and Darnhofer 2015).

The access to the Kuwalla communal pasture as well as its management rules are governed by an informal institution that includes a management committee, ‘father of herders,’ and a general assembly. The committee is composed of four members, all of whom are men. They are responsible for overseeing the implementation and revision of the rules-in-use. The committee is backed up by nine ‘father of herders,’ each of which is responsible for a sub-group of users. The role of the ‘father of herders’ is to coordinate and facilitate the implementation of the rules by conveying information from the committee to their sub-group of users. The rules that are communicated include which paddock is to be grazed when, and whose turn it is to guard the pasture against trespassers. The management committee and the ‘father of herders’ are elected by users every 2–4 years in the general assembly. The general assembly is attended by the head of the households.

**Fig. 3:**
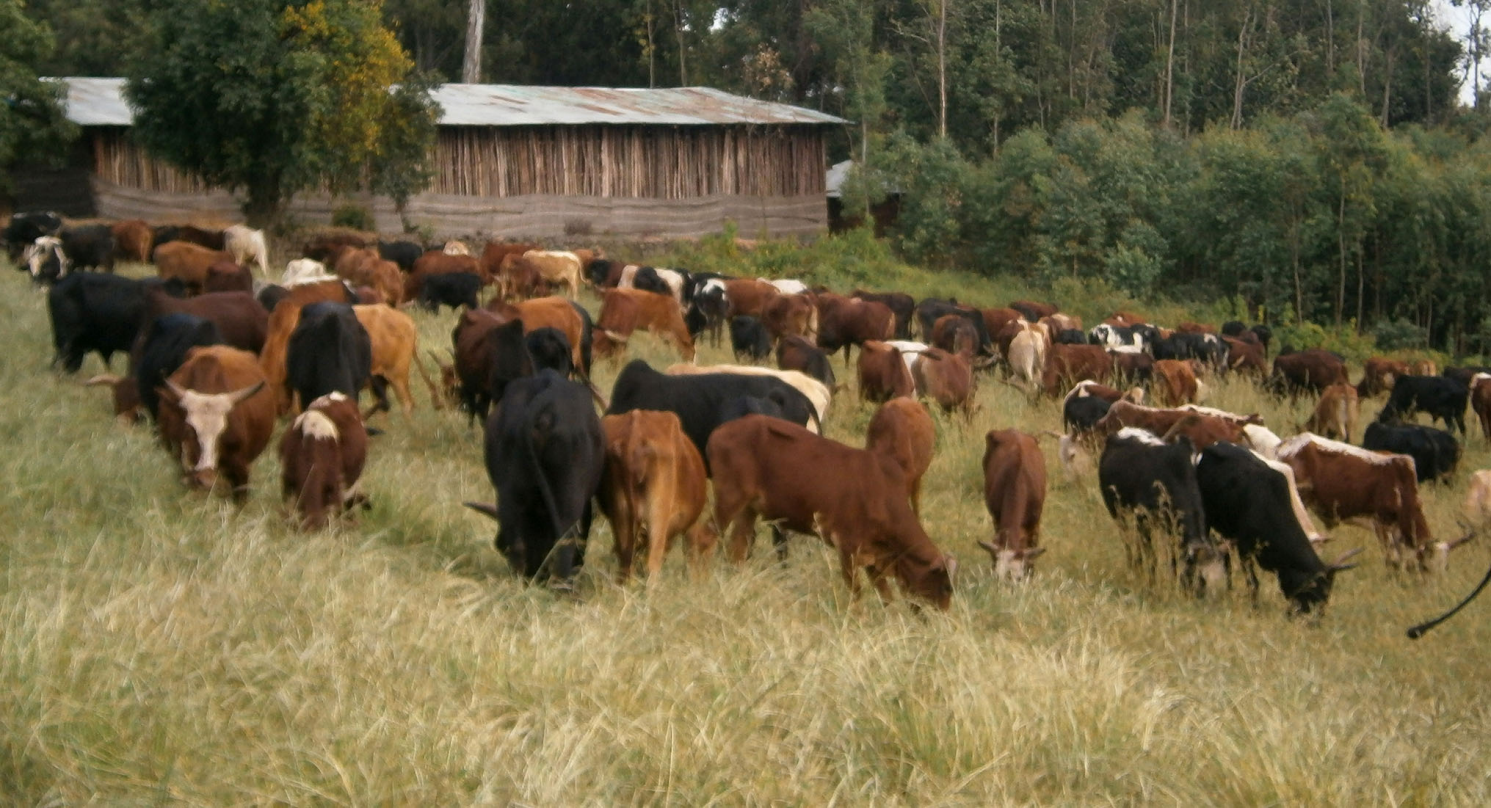
Cattle grazing on the communal pasture in Kuwalla. Only a relatively small paddock is opened for grazing each day to ensure that the available grass is well used and to avoid trampling of grass, which would waste feed resources. The communal pasture is only for grazing cattle, as sheep and other farm animals are excluded. While this protects the pasture from overstocking, it also excludes poor households, i.e., those not owning any cattle

Since its inception in 1990, the informal institution has established a sophisticated rotational grazing system that has ensured feed availability throughout the year. The communal pasture is grazed only in certain periods of the year (between April and July, and in October). During the two opening seasons, the pasture is divided into paddocks. Cattle graze in one paddock for a day and then move to the next paddock the following day to avoid overgrazing and to ensure an equal spread of the dung (Fig. 3). Grazing priority is given to oxen, as oxen tend to be seen as the most important type cattle, since they are needed as drought power to plough fields. Each household is thus allowed to send all its oxen to the communal pasture (up to five, as no household in Kuwalla owns more than five oxen). Households which do not own oxen can send up to two cows, heifers, bulls, or calves. Other animals such as sheep (*Ovis aries*), goats (*Capra aegagrus hircus*), donkey (*Equus asinus*), and horses (*Equus ferus caballus*) are not allowed to graze on the communal pasture. They are only allowed to graze on the free grazing area, which is accessible to all animals, year-round.

### Data collection and analysis

A qualitative case study approach (Yin 2003; Nightingale 2006) was used to collect data on gender relations and the management of the communal pasture. This allowed an in-depth exploration of how socially defined gender roles and norms are linked to the choices by the community on how to manage the communal pasture. Moreover, it allowed to explore the different views and experiences of men and women regarding the current management rules, and to capture the voices of marginalized groups, such as the women and the poor (Sally and Fonow 2012). A mix of qualitative data collection methods was used: focus group discussions, in-depth interviews with key informants, participant observation, and a reflection meeting with community and research stakeholders. This mix of methods helped to triangulate and cross-check the information collected, thus enhancing the validity and reliability of the results (Yin 2003; Sally and Fonow 2012).

Data were gathered in two periods (September–December 2012 and September–October 2013). To capture the diversity of views, a total of 11 focus group discussions with four distinct groups were conducted: a core group which included elders, youths, poor, rich, men, and women; a group with the current management committee and the ‘father of herders’; a group with men only; and a group with women only. Each group comprised 6–10 villagers which were selected based on their familiarity with the discussion topics. Additionally, interviews were conducted with 14 key informants from the community (seven men and seven women), as well as with seven experts, from the District Office of Agriculture and the District Office of Land Administration and Use. All focus group discussions and interviews were held in Amharic. The first author translated and transcribed relevant sections of the discussions and of the interviews into English.

The data were analyzed using qualitative content analysis (Berg 2009). The partial transcripts were coded using the qualitative data analysis software ATLAS ti (version 7.0.06), based on pre-defined concepts (Brayman and Burgess 2005). The initial codes included: changes, knowledge, adaptation, social learning, collective action, programs, and social norms. The social norms were further sub-coded into gendered needs, gendered roles, and gendered knowledge, and guided the comparative analysis between the men’s and women’s interviews. Additional codes were defined during the analysis, e.g., incentive, negotiation, trust, social network, leadership, and conflict (for details, see Aregu 2014). The aim of the analysis was to characterize and contrast the roles, needs, and knowledge of men and women in the management of the communal pasture and to understand their perception of the benefits and drawbacks regarding the current management arrangements.

The preliminary findings were presented at three reflection meetings to research participants at community level, and to experts at district and national levels. The aim was to ensure that the data and findings accurately reflected the description given by the research participants, thus helping to ensure the validity of the data. Moreover, sharing the preliminary findings contributed to reflective learning through raising the awareness of stakeholders about the importance of understanding the influence of gender relations on the management of the communal pasture.

## Results and discussion

We first describe women’s position in the informal institution governing the use and management of the communal pasture in Kuwalla. Subsequently, we analyze how not taking into accounts gendered needs, preferences, and knowledge may undermine the resilience of the communal pasture.

### Gendered roles: Influence on the informal institution

The communal pasture in Kuwalla is widely perceived as well-managed by experts, given that the management rules have successfully avoided overstocking, overgrazing, and soil erosion. However, the benefits derived from the communal pasture are not evenly distributed between men and women, nor between rich and poor households. Indeed, rich households (i.e., those who own more than two oxen) benefit most as they can send all of their oxen. Poor households (i.e., those who do not own cattle) do not directly benefit from the communal pasture, as animals such as sheep are not allowed to graze on it. This exclusion disproportionally affects women, as 63 % of poor households are headed by women. Even in rich households, married women often cannot send their cows to pasture, as their husbands tend to argue that it is better to send three or more oxen, rather than just two cows (see details on page 82 in Aregu 2014).

**Fig. 4:**
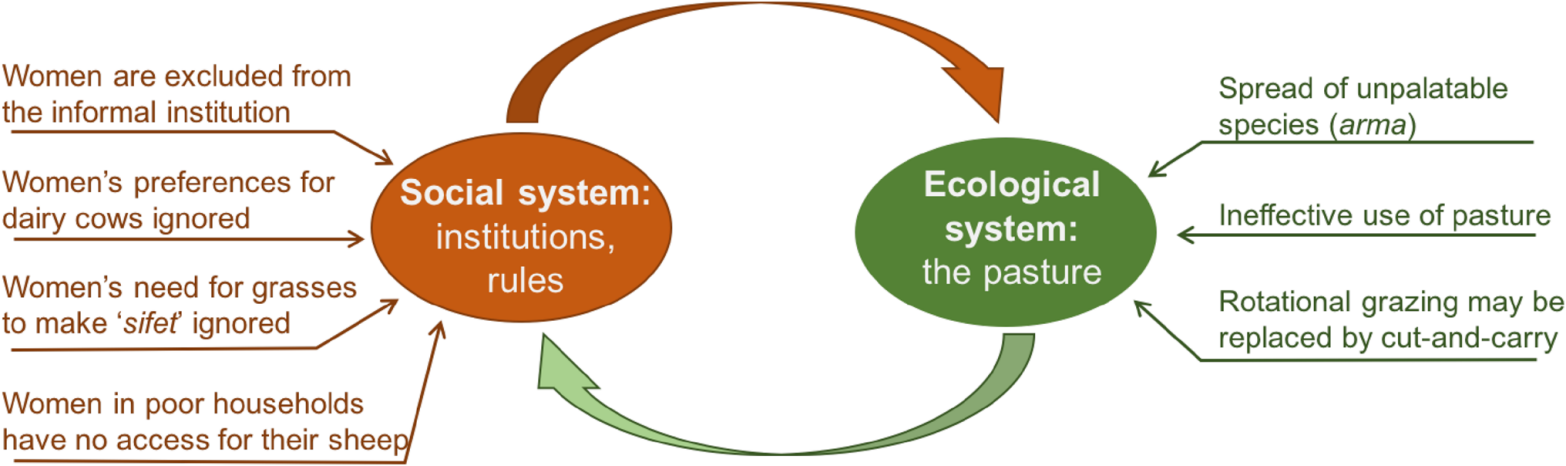
Gender analysis shows how social dynamics, driven by socially defined gender roles, may affect the management of the ecological system. The resilience of the social-ecological system can be weakened if the needs of women and of poor households are ignored, and if women’s knowledge is not taken into account. The needs and preferences of various social groups are dynamic, not least in response to changes in the broader context. This highlights the importance of inclusive social learning as a driver to adapt management rules, rather than a one-sided focus on ecological indicators as cues for the need to change management practices

The management rules thus perpetuate gender inequalities and the marginalization of poor households. Inequalities and marginalization can be challenged both from an ethical point of view (Leach et al. 2012), and from an ecological point of view. Indeed, the social and ecological sub-systems are inseparable and tensions in the social system are very likely to affect the management and thus the sustainability of ecological system (Fig. 4).

Rules and their evolution are usually driven by those voices that are represented (Leach et al. 2010). Whose interests are acted upon, is the outcome of negotiations (Eriksen and Brown 2011). In Kuwalla, the informal institution that governs the communal pasture is controlled by men. Indeed, women are not involved in decision-making in the informal institution as the management committee has never had a member that was a woman since its inception in 1990. Similarly, all ‘fathers of herders’ are and always have been men. While the general assembly is attended by some women (those who are heading a household), they do not usually participate actively. Women from male-headed households have few opportunities to express their views and preferences in the process of crafting the rules-in-use during general assemblies, as they are represented by their husbands. Husbands may or may not take their wives’ ideas and suggestions into account during discussions on the management rules (Agarwal 2001; Giri and Darnhofer 2010).

The informal institution is an important platform to negotiate, learn, and adapt the management and access rules. Effectively excluding women from it is not only depriving them of the opportunity to directly influence the rules, but also deprives the committee members and the general assembly members of the opportunity to hear the women’s ideas and to listen to their concerns.

This has meant that the women’s preference to balance the grazing of oxen and of lactating cows has remained unheard; that the need of women from poor households to secure feed for their sheep has been ignored; and that women’s knowledge regarding grass species has been overlooked. Disregarding these needs and preferences has affected the communal pasture in two ways. Firstly, as oxen are systematically privileged, dairy cows receive less protein-rich feed than would be possible, thus reducing their milk yield. Secondly, women have been banned from harvesting a specific grass species they need to craft household items. As a consequence, discontent has been growing, which undermines the legitimacy of the informal institution and thus threatens the resilience of the social-ecological system.

### Gendered preferences: Lactating cows versus oxen

Traditionally, women are responsible for the care of cows and they control the income from milk products (butter and cheese). Women are thus interested in providing sufficient and high-quality feed to their lactating cows. An increase in milk production would allow them to improve the quantity of food available to their family and to increase their cash income. Given their interests, women have a nuanced knowledge about the feed species that increase milk production. Indeed, when men and women were asked to list plant species found on the pasture and assess their importance, the women ranked *mesobei* (*Medicago polymorpha*) and *wajima* (*Trifolium* spp.) higher than the men. The women were well aware that these species are protein-rich and increase milk production. While men knew these species, they did not rank them as particularly important (for details, see page 68 in Aregu 2014).

However, the current rules constrain women’s ability to send their cows to graze on the communal pasture and to benefit from protein-rich feed. Indeed, the access rules privilege oxen, as each household can send all its oxen to the communal pasture (up to five, as no household in Kuwalla owns more than five oxen). Households which own two oxen can send them both; households which own one ox can send an ox and a cow; households which do not own any oxen can send up to two cows. Thus, while theoretically, the choice whether to send oxen or cows to the pasture is a matter of intra-household decision-making, in practice, only women in households with one or no ox are able to send one or two cows to the pasture. This leads to two problems: the women perceive current rules as unduly privileging men’s interests, and the use of the feed resources is sub-optimal.

During the focus group discussions and during individual interviews women expressed their disappointment that their cows, particularly when they were lactating, were in effect being denied access to the communal pasture. One woman expressed this wish for a more balanced access rules for the cows (key informant interview, Nov. 2012):

“Even though oxen are important to the family for crop cultivation and to get what we eat, cows should not be neglected. They are important to have milk for our children, to get butter as source of income for us [women], and to give the future oxen to the family.”

It would seem desirable that lactating cows get priority access to the pasture, especially outside the ploughing period when oxen do not need protein-rich feed. Providing cows access to protein-rich *mesobei* and *wajima,* would allow increasing milk production without any drawback for the oxen. Yet, the gender-biased rules systematically privilege oxen over dairy cows. This rule mirrors prevailing social values. Indeed, beyond the importance of oxen for crop cultivation, oxen also convey social status: the more oxen are owned by a household, the wealthier the household is considered. Moreover, men derive personal prestige when they own strong and beautiful oxen. They are then widely seen as ‘good’ farmers by the community, and thus as deserving respect. These social values reinforce men’s preference to provide privileged grazing access to oxen.

This example shows that women and men tend to have different preferences linked to their gendered social roles (securing milk supply for the family versus ploughing fields with oxen; income from milk products versus status and prestige from well-fed, beautiful oxen); these preferences lead to different knowledge about grass species, e.g., those that are particularly protein-rich. Yet, as a result of excluding women from the management committee and marginalizing them during general assemblies, their preferences and their suggestions to optimize the use of scarce feed resources are not taken into account. Marginalizing women can thus hamper the open discussion of relative merits of different rules and thus the discussion about options to adapt management rules. Yet, such adaptation can be required, e.g., to respond to demographic dynamics, to ensure that the changing constraints and needs of various social groups are taken into consideration, and to take into account shifts in what is perceived as a fair distribution of scarce resources.

### Gendered needs: Sheep versus cattle

Gendered social roles, such as the responsibility for specific livestock categories, shape the needs of men and women and thus their preferred use of the communal pasture. Sheep are an important asset for women in both female-headed and male-headed households, as women control the income from the sale of sheep. Moreover, sheep are often the only livestock asset owned by female-headed households, the majority of which are poor. As a result, women have an interest in gaining access to the communal pasture for their sheep.

However, current rules prevent sheep from entering the communal pasture, which means that women from poor households have no direct benefits from the communal pasture. As a result, their support for the current rotational grazing management system is waning. They start to question the legitimacy of the informal institution, and increasingly support a switch to a cut-and-carry system (woman heading a household, key informant interview, Oct. 2012):

“It would be have been good to use the pasture through the cut-and-carry system. (…) I could also get some pasture to fatten the sheep and sell them for a good price during festivals.”

In a cut-and-carry system, the pasture is completely closed year-round and can only be accessed to cut the grass by hand, to feed the cattle elsewhere. This approach is heavily promoted by the District Office of Agriculture (expert from DOA, key informant interview, Dec. 2012):

“We want the Kuwalla community to adopt the cut-and-carry system, because the households who do not own cattle can get their share of the feed through cutting. They can either feed it to their sheep or they can sell it.”

The management committee is thus under pressure both internally from women and poor households generally, and externally from experts from the District Office of Agriculture. Given the current rate of population growth in Ethiopia, the limited land resources, and the unequal distribution of the land (Bielli et al. 2011), the number of poor households is likely to increase. The number of households headed by women is also likely to increase, as many men migrate to cities in search of employment (Regassa and Yusufe 2009; Gibson and Gurmu 2012). These broader social dynamics might well reinforce the emerging internal and external pressures that challenge the informal institution and thereby undermine the current arrangements to manage the communal pasture. Thus, unless the informal institution addresses the needs of marginalized households, especially of poor, female-headed households, it is likely that its legitimacy will increasingly be questioned.

This pressure from marginalized groups is a source of stress, threatening the sustainability of the whole management system (Ostrom 1990). While the informal institution has adapted rules in the past (Aregu and Darnhofer 2015), it currently does not demonstrate its capacity to address emergent dynamics, which might indicate a limited adaptive capacity, which weakens the resilience of the social-ecological system.

### Gendered knowledge: Alternative use of grasses

Another example illustrating the impact of gendered roles on preferred pasture management practices is linked to one of the women’s roles in the household. Traditionally, women are expected to craft *sifet*, a basket and a basic household utensil used to serve and store food. A woman who is good at making *sifet* is traditionally considered a ‘good’ wife. To make *sifet*, women need to collect two grass species: *zeba* (*Hyparrhenia dregeana*) and *arma* (*Eleusine floccifolia*). While these two grasses are commonly found on the communal pasture, women are not allowed to collect them, as the communal pasture is to be used exclusively to graze cattle. The women thus have to purchase the grasses at the market. Yet, women are often unable to purchase *arma* at the market, so they turn to a plastic thread locally known as *madaberia*, named after the bag used to transport and store fertilizer. According to the women focus group, *sifet* made using *madaberia* is not suitable to serve and keep hot food. Thus, while collecting the grasses to make *sifet* from the pasture is unlikely to significantly reduce the amount of feed available to the cattle as the quantities needed are limited, women’s need for *arma* has not been discussed by the committee members.

The prohibition to cut *arma* is ironic, as the management committee highlighted *arma* as one of the species that is threatening the quality of feed resources due to its abundance. Indeed, as the grass matures and dries, it is no longer palatable and thus not grazed by cattle. Yet, the men in the management committee never thought of giving women access to harvest the grass, a measure that could contribute to controlling its spread and thereby maintain the quality of the pasture. Instead, they have searched for ways to control the spread of *arma* (man from the management focus group discussion, Oct. 2012):

“We asked the experts to tell us if there are any herbicides that kill it. But we learned from them that they would also kill other grass species. So we were afraid of using herbicides and so we did not try any (…). Since last year, we have been uprooting a few of them, but this grass still keeps spreading every year.”

The women’s need for the grasses should be known to the men involved in the informal institution, as despite strong rules making the harvesting of grass illegal, some women steal *arma* and *zeba*. As one key informant noted (woman, key informant interview, Oct. 2012):

“My daughter used to steal zeba from the controlled communal pasture. She was caught once but got out of it before it was reported to the management body where she would have had to pay a fine. (…) The guard realized that the amount she took was too small.”

This behavior illustrates what Agarwal (1997a) and Leach et al. (1999) predict: if rules are perceived as unfair, resource users may question the legitimacy of the rules governing the management of natural resources.”

If women’s needs had been heard and taken seriously by the management committee, if the women had been invited to actively take part in a discussion on how to address the problem caused by the increasing abundance of *arma*, it would have been likely that a win–win situation could have been identified, leading to a change in access and use rules. This adaptation of rules could have satisfied the women’s need to harvest *arma* to craft their household items, the cattle’s need for sufficient feed, and the community’s need to maintain the quality of the pasture. This demonstrates Scheffer’s and Westley’s (2007) argument that if social structures and institutions remain rigid, available knowledge will not be integrated. By excluding women, social learning was impaired and needed changes were not implemented. Overall this reduced the adaptive capacity of the social-ecological system, and thus its resilience.

## Conclusion

The case study of the communal pasture in Kuwalla was specifically selected as it is widely acknowledged to have been managed sustainably, whereas most other pastures in the Ethiopian highlands are severely degraded. A gender analysis of the management rules shows that these are highly biased against the priorities of women and of poor households, most of which are headed by women. The rules for the management of the communal pasture are biased toward men’s values, enabling them to fulfill their traditional roles and responsibilities in the management of livestock. They thus mirror men’s preferences (for beautiful, well-fed oxen) and needs (strong oxen for ploughing), as well as build on men’s knowledge of grass species. As a result of their marginalization, many women—especially those of poor households—do not derive any direct benefit from the communal pasture. As a result, they have started to question the legitimacy of the informal institution and to undermine its rules. Indeed, inequality between groups of users is bound to generate social resentment and disincentives to comply with management rules (Agarwal 2001; Andersson and Agrawal 2011).

This study thus underlines the importance of taking the gender-dimension into account when considering how to strengthen the adaptive capacity of a social-ecological system, thus strengthening its resilience. Change is inevitable and both drivers and solutions are never gender-neutral. Gender-blindness is problematic: ignoring women’s needs, preferences, and knowledge when designing or revising management rules has undermined the ability of the informal institution to address two important social and ecological challenges. First, preventing women and poor households from feeding their dairy cows and sheep perpetuated inequality in the community, possibly increasing poverty. Second, the spread of a poor-quality grass species reduces the quality of the communal pasture, which affects the whole community. Both developments risk undermining the legitimacy of the informal institution, and thus threaten the whole management system. This threat is even more salient as the system is under external pressure, given that the District Office of Agriculture favors a cut-and-carry approach over rotational grazing.

Including women in the decision-making process, and increasing the gender-dimension of many management choices, may provide the opportunity to enhance resilience in two ways. First, it can strengthen the ability of the community to take effective steps in adapting to change through fostering the exploration of a diversity of options, based on diverse interests of rich and poor, and knowledges of men and women. Openly exploring diverse opportunities and gauging trade-offs can enhance social learning in the community. Second, it can contribute to identifying ways that allow women and poor households to benefit from the communal pasture, enhancing gender equality and social justice (Leach et al. 2012). Indeed, social equity and social justice issues are key aspects in the resilience of social-ecological systems (Eriksen and Brown 2011; Wuelser et al. 2012; Brown 2014). As the Kuwalla case study illustrates, a gender blind approach to resilience is likely to overlook important differences in preferences, needs, and knowledge between men and women. Indeed, socially defined gender roles play an important role in structuring responsibilities, in the participation in decision-making processes, and in the access to resources in many communities whose livelihood is directly dependent on natural resources.

## Footnotes

1 A *kebele* is the smallest formal administrative unit in Ethiopia.
